# The Book World of Medicine and Science

**Published:** 1897-03-27

**Authors:** 


### The Book World of Medicine and Science.

Practical Manual of Diseases of Women and Uterine Therapeutics. By H. Macnaughten Jones, M.D. Seventh edition. Revised and enlarged, with 565 illustrations. (London: Baillière, Tindall, and Cox. 1897. Price 16s.)

A seventh edition ought not to require much reviewing. In the production of this edition, however, it is clear that the book has undergone a very careful revision, and although the old characteristics are retained it comes before us in its new form as a very up-to-date and complete summary of modern knowledge in the departments of medicine and surgery of which it treats. Its arrangement is systematic, and reference to its contents is made easy by a fairly complete index; it is well illustrated, is full of information, and will no doubt be found a very useful repertory of knowledge by many a practitioner. As a guide to practice, however, it is a book that will have to be used with a certain amount of caution. By careful reading it is easy enough to find out what the author recommends and what he does not; but unless the reader bears in mind that the book describes a great deal of which the author disapproves he might be misled into adopting treatment which although mentioned is not endorsed. For example, paracentesis abdominis, vaginal punction (*sic*), and vaginal aspiration or tapping of a pelvic hæmatocele are all described, although their general inappropriateness is insisted on. In the same way, a long string of substances is given, including many fluids which may be used for intra-uterine medication, the sort of syringe or injector to be employed is discussed, and the precautions necessary in the performance of the operation are described. Nevertheless, we are told with all the emphasis given by italics, "*I never resort to intra-uterine medicated injections into the cavity of the uterus*," and later on "I repeat that in practice I believe intra-uterine injection to be a needlessly venturesome plan of treating unhealthy endometric conditions." The book is evidently a reservoir of knowledge, besides being a guide to treatment. Many hard things are said of pessaries, but all the same many pessaries are described and figured. All this, however, is useful if the book is carefully read. As a guide to the student we think the book would bear a good deal of pruning, but, as a handbook for the use of the practitioner, we have no doubt that it will be found of considerable utility.

